# New insights on the cardiovascular effects of IGF-1

**DOI:** 10.3389/fendo.2023.1142644

**Published:** 2023-02-09

**Authors:** Mirjana Macvanin, Zoran Gluvic, Jelena Radovanovic, Magbubah Essack, Xin Gao, Esma R. Isenovic

**Affiliations:** ^1^ Department of Radiobiology and Molecular Genetics, VINČA Institute of Nuclear Sciences - National Institute of the Republic of Serbia, University of Belgrade, Belgrade, Serbia; ^2^ Clinic for Internal Medicine, Department of Endocrinology and Diabetes, Zemun Clinical Hospital, School of Medicine, University of Belgrade, Belgrade, Serbia; ^3^ Computational Bioscience Research Center (CBRC), King Abdullah University of Science and Technology (KAUST), Thuwal, Saudi Arabia; ^4^ Computer Science Program, Computer, Electrical and Mathematical Sciences and Engineering Division (CEMSE), King Abdullah University of Science and Technology (KAUST), Thuwal, Saudi Arabia

**Keywords:** IGF-1, cardiovascular diseases, IGF-1 system, atherosclerosis, diabetes, microRNA

## Abstract

**Introduction:**

Cardiovascular (CV) disorders are steadily increasing, making them the world’s most prevalent health issue. New research highlights the importance of insulin-like growth factor 1 (IGF-1) for maintaining CV health

**Methods:**

We searched PubMed and MEDLINE for English and non-English articles with English abstracts published between 1957 (when the first report on IGF-1 identification was published) and 2022. The top search terms were: IGF-1, cardiovascular disease, IGF-1 receptors, IGF-1 and microRNAs, therapeutic interventions with IGF-1, IGF-1 and diabetes, IGF-1 and cardiovascular disease. The search retrieved original peer-reviewed articles, which were further analyzed, focusing on the role of IGF-1 in pathophysiological conditions. We specifically focused on including the most recent findings published in the past five years.

**Results:**

IGF-1, an anabolic growth factor, regulates cell division, proliferation, and survival. In addition to its well-known growth-promoting and metabolic effects, there is mounting evidence that IGF-1 plays a specialized role in the complex activities that underpin CV function. IGF-1 promotes cardiac development and improves cardiac output, stroke volume, contractility, and ejection fraction. Furthermore, IGF-1 mediates many growth hormones (GH) actions. IGF-1 stimulates contractility and tissue remodeling in humans to improve heart function after myocardial infarction. IGF-1 also improves the lipid profile, lowers insulin levels, increases insulin sensitivity, and promotes glucose metabolism. These findings point to the intriguing medicinal potential of IGF-1. Human studies associate low serum levels of free or total IGF-1 with an increased risk of CV and cerebrovascular illness. Extensive human trials are being conducted to investigate the therapeutic efficacy and outcomes of IGF-1-related therapy.

**Discussion:**

We anticipate the development of novel IGF-1-related therapy with minimal side effects. This review discusses recent findings on the role of IGF-1 in the cardiovascular (CVD) system, including both normal and pathological conditions. We also discuss progress in therapeutic interventions aimed at targeting the IGF axis and provide insights into the epigenetic regulation of IGF-1 mediated by microRNAs.

## 1 Introduction

Cardiovascular diseases (CVDs) are the leading cause of morbidity and mortality globally, accounting for 32% of all deaths (1). Coronary artery disease (CAD) is the most common comorbidity, and severe complications of this vascular pathology are heart attack, arrhythmias, chronic nephropathy, and ischemic stroke (2, 3). Peripheral artery disease (PAD), also characterized by atherosclerotic plaque formation, manifests as lesions and pain in the lower extremities (4, 5). Other heart and blood vessel disorders include deep vein thrombosis, pulmonary embolism, and rheumatic and congenital heart disease (1, 6, 7). The principal risk factors for CVDs are genetic predispositions, metabolic syndrome (MetS), inadequate dietary and lifestyle habits, and physical inactivity (8). Insulin resistance, diabetes mellitus (DM), dyslipidemia, elevated blood pressure, and visceral and abdominal obesity represent predisposing factors for the MetS occurrence, which strongly correlates with cerebral and abdominal aneurysms, CAD, PAD, and stroke (9, 10).

Insulin-like growth factor-1 (IGF-1) is a polypeptide growth factor with a structure comparable to insulin. IGF-1 is responsible for cell differentiation, maturation, growth, and proliferation in almost all body organs (11–13). Numerous reports advocate the hypothesis that IGF-1 participates in the homeostasis of cardiovascular (CV) physiology. In particular, IGF-1’s roles in the CV system include maintaining cellular homeostasis by regulating vascular vasoconstriction/vasodilatation, cardiac apoptosis and autophagy, and inflammatory responses (14–21). Furthermore, IGF-1 exerts significant anti-inflammatory and anti-oxidant effects on the vasculature, decreasing atherosclerotic plaque burden (22). In addition, IGF-1 possesses anti-atherogenic properties reflected in its role in modulating endothelial junction protein levels and promoting angiogenesis in endothelial cells (ECs) (23, 24). With the rising prevalence of CVDs, IGF-1’s involvement in CV system functioning is attracting substantial research interest. This review discusses recent research on the roles of IGF-1 in physiological and pathological conditions such as CV and metabolic diseases. We review IGF-1’s roles in atherosclerosis, CAD, PAD, hypertension, and diabetes. Furthermore, we discuss progress in therapeutic interventions that target the IGF axis and provide novel insights into the epigenetic regulation of IGF-1 mediated by microRNAs (miRNAs).

## 2 IGF-1 system

**Figure 1:**
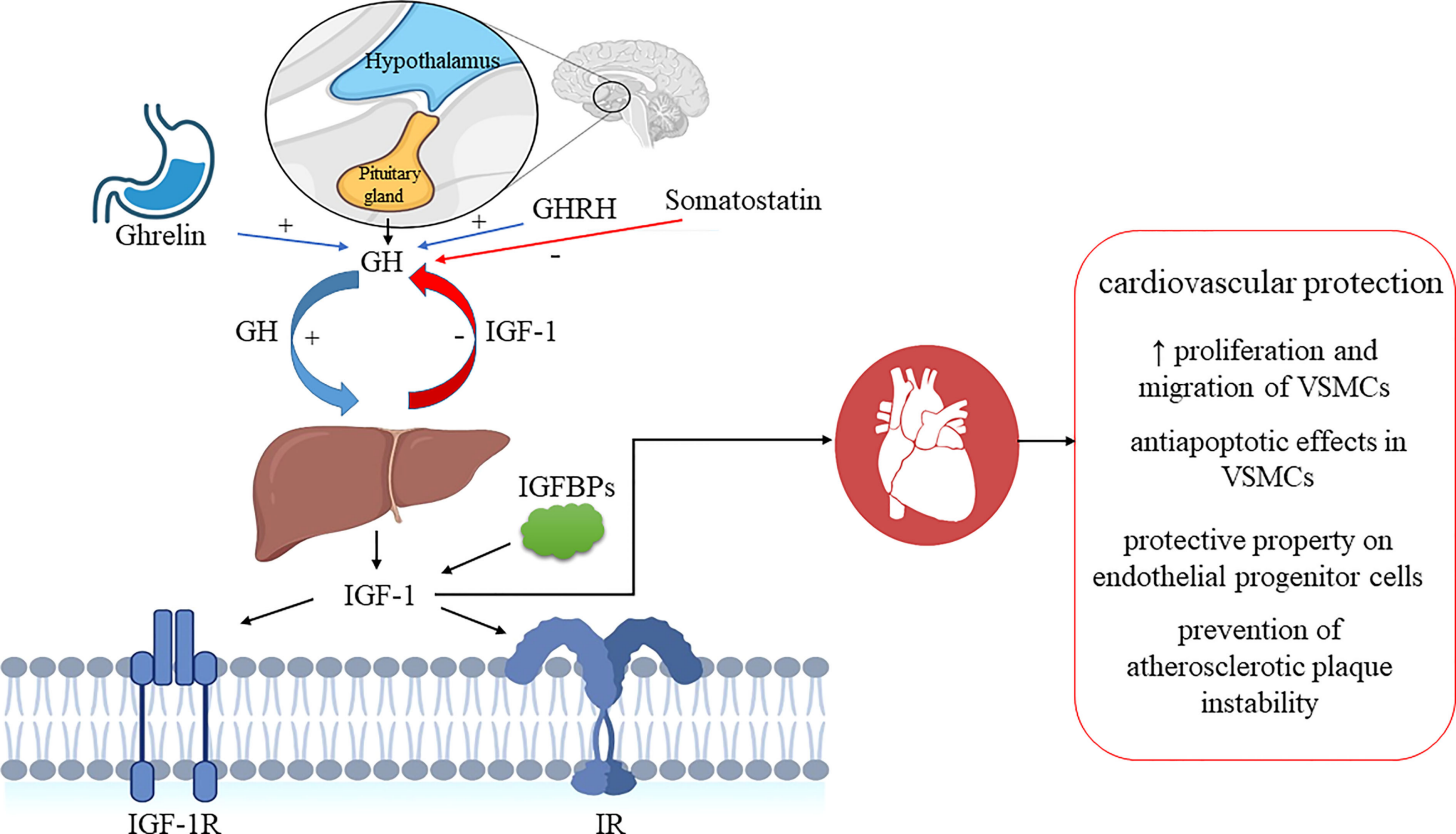
IGF-1 system and GH/IGF-1 axis regulation. Ghrelin and GHRH stimulate pituitary secretion of GH, while somatostatin inhibits it. GH stimulates IGF1 secretion in the liver, and IGF-1 exerts negative feedback and inhibits GH secretion. GH, growth hormone; GHRH, growth hormone-releasing hormone; IGF-1, insulin-like growth factor-1; IGF-1R, insulin-like growth factor receptor-1; IGFBPs, insulin-like growth factor binding proteins; IR, insulin receptor; VSMCs, vascular smooth muscle cells. Biorender.com was used to generate part of the Figure.

**Figure 2:**
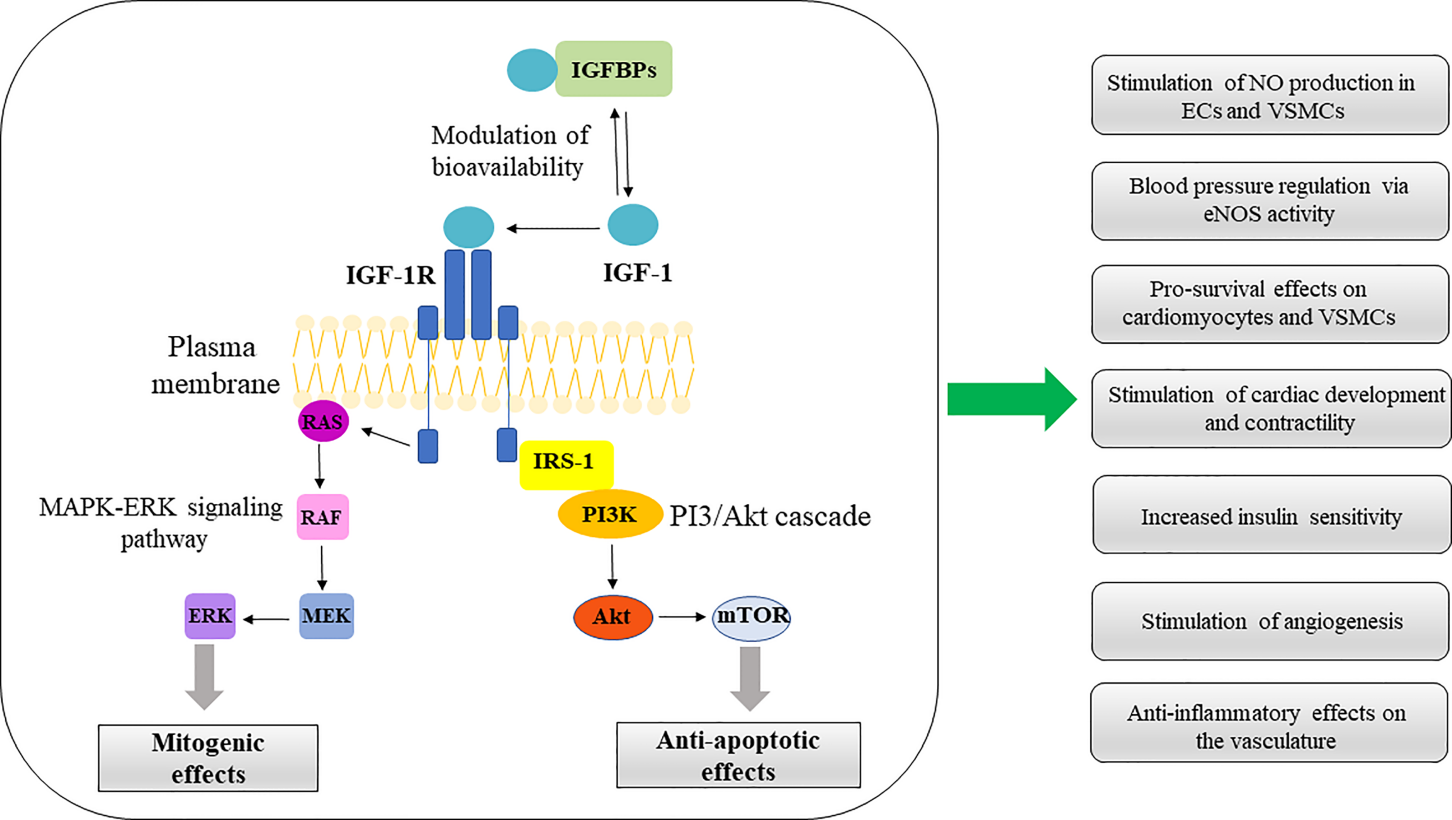
IGF-1 signaling pathways in CV system. ERK/MAPK and PI3K/Akt represent the main pathways involved in IGF-1 signal transduction. Activation of these pathways confer multiple protective effects on the CV system, summarized in the diagram. IGFBPs, insulin-like growth factor binding proteins; IGF-1, insulin-like growth factor-1; IGF-1R, insulin-like growth factor receptor-1; RAS, rat sarcoma protein; RAF, rapidly accelerated fibrosarcoma kinase; MEK, mitogen-activated protein kinase kinase; ERK, extracellular signal-regulated kinase; IRS1, Insulin receptor substrate-1; PI3K, phosphatidylinositol-3 kinase; Akt, serine/threonine kinase (protein kinase B); mTOR, mammalian target of rapamycin; NO, nitric oxide; VSMCs, vascular smooth muscle cells; eNOS, endothelial nitric oxide synthase.

Salmon and Daughaday (25) discovered IGF-1 in 1957 and initially described it as a “sulfation factor” that stimulated the incorporation of ^35^Sulfate by costal cartilage. In 1976 it was renamed “insulin growth factor-1” by Rinderknecht and Humbel to reflect its structural resemblance to proinsulin (26). IGF-1 contains 70 amino acids (AAs), has a molecular weight of 7.649 kDa and belongs to a family of insulin-related single-chain peptides (27, 28). The IGF-1 coding gene is on chromosome 12 in humans (29). IGF-1 possesses three disulfide bridges (between AAs 6 and 48; 18 and 61; 47 and 52) and binds to both IGF-1 receptor (IGF-1R) (with a high affinity) and insulin receptors (IR) (with a low-affinity) (30). IGF-1 is mainly synthesized in the liver and exhibits endocrine, paracrine, and autocrine activity (31). As the primary mediator of the anabolic and mitogenic activity of growth hormone (GH), IGF-1 plays a significant role in cellular physiology in childhood and adolescence (32, 33). The primary IGF-1 role is its endocrine activity, but it can also be secreted by tissues other than the liver, in which it acts locally and expresses a paracrine function (12, 34–36). IGF-1 is a negative regulator of pituitary GH production (**Figure 1**) (35). IGF-1R is present in almost every cell in the organism, and it contains two alpha and two beta subunits associated with disulfide bonds. Beta subunits have tyrosine kinase domains, whose activation is mediated by the binding of IGF-1 to alpha subunits, and it is associated with various signaling pathways, including PI3K/Akt and Raf/MEK/ERK cascade (37, 38) (**Figure 2**). The IGF-1R gene is positioned on chromosome 15q26 and has approximately 70% similarity with the IR gene (39, 40). In addition to IGF-1R, insulin-like growth factor binding proteins (IGFBPs) are required for IGF-1 bioavailability because they interact with IGF-1 with the same affinity as IGF-1R (41, 42). There are six IGFBPs types (containing approximately 200–300 AAs) that regulate IGF signaling and prolong its half-life, and they bind more than 90% of IGF-1 present in circulation (about 1% of IGF-1 in circulation are in a free-active form), simultaneously blocking its binding to IR (42, 43). Among all known IGFBPs, the most abundant is IGFBP-3, with a concentration of 100 nM/L in adult serum, and represents an essential protein in the IGF pathway. The IGF-1/IGFBP-3 ratio is used as a clinical predictor of metabolic syndrome (MetS) (11, 44). It is assumed that IGFBPs could act in either IGF-dependent or independent manner, and due to different IGFBP expression profiles in various tissues, they are investigated as diagnostic biomarkers and potential therapeutic agents for different pathological disorders (45–47).

### 2.1 Roles of IGF-1, IGF-1R, and IGFBPs in vascular smooth and endothelial cells

IGF-1 and IGF-1R are expressed by vascular smooth muscle cells (VSMCs), endothelial cells (ECs), and macrophages (34), and together with different IGFBPs, they are considered significant regulators of atherosclerosis pathophysiology whose expression is downregulated in atherosclerotic lesions (48–50). IGF-1 participates in the proliferation and migration of VSMCs, and according to numerous data, decreased IGF-1 and IGFBPs serum levels have been associated with carotid atherosclerosis (51–53). In addition, decreased circulating levels of IGF-1 and IGFBP3 have been correlated with a greater incidence of ischemic heart and cerebrovascular stroke (53, 54). In a recent case-control study with more than 4000 participants, it was proposed that the IGF-1 signaling pathway contributes to the risk of stroke occurrence (55). Atherosclerotic plaque stability and dimensions are partly regulated by VSMCs, whose apoptosis and structural alterations influence plaque rupture (56–58). Interestingly, one of the proposed IGF-1 mechanisms in the context of CVDs is the prevention of atherosclerotic plaque instability through VSMCs phenotype modulation and anti-apoptotic effects (59, 60). IGF-1 gene expression in VSMCs is dependent on numerous factors, including thrombin, tumour necrosis factor-alpha (TNFα), angiotensin II, oxidized low-density lipoprotein (ox-LDL), and reactive oxidative species (ROS) (61–66). As previously mentioned, IGF-1 bioavailability is controlled through six IGFBPs. For instance, IGFBP4 is the principal IGFBP produced by VSMCs, and its decreased expression in VSMCs may increase IGF-1 bioavailability (67). IGFBP-1 affected SMC proliferation and was implicated in regulating plaque stability (68). Furthermore, IGFBP2 enhances endothelial cell chemotaxis (69), while IGFBP3 is related to regenerative potential in cardiac atrial appendage stem cells (70). Endothelial dysfunction is an important part of CVDs pathologies and related comorbidities, such as atherosclerosis, hypertension, diabetes, and obesity development and prognosis, as endothelium manages vascular homeostasis (71). IGF-1R moderates EC function through its impact on nitric oxide (NO) bioavailability since it can form hybrid receptors with the insulin receptor (IR) and consequently influence endothelial cell sensitivity to insulin (72). In the rat model of acute renal failure, IGF-1 demonstrated NO-mediated ameliorative action on renal function, possibly due to NO’s vasodilatation effects (73). Also, IGF-1 manifests a protective property on endothelial progenitor cells by preventing its dysfunction caused by oxLDLs *via* eNOS/NO signaling pathway (74). In addition, one of the proposed novel IGF-1 protective features regarding endothelial function is the upregulation of endothelial junction protein levels (24).

According to the literature data, IGF-1 participates in the regulation of peripheral resistance (75). This important growth factor contributes to pressure regulation since it increases blood flow in rats through vasodilatation, and hypertension has been observed in IGF-1 genetically deficient mice (76, 77). New findings suggest that matrix metalloproteinases receptor cleavage has a notable role in the ineffective IGF-1 signaling in hypertension and might be significant for peripheral vascular resistance (19). Interestingly, manipulation of IGF-1 levels in the peripheral circulation and its increased levels in the brain could be used to recover early manifestations of cerebral artery occlusion (78). Regarding human studies, IGF-1 infusion reduced peripheral resistance in patients with chronic heart failure, suggesting its acute impact on the CV system (79). Besides, IGF-1 serum and plasma levels in humans are considered valuable biomarkers for CVD development. For instance, low serum IGF-1 is linked to hypertension and early CV complications in female patients with rheumatoid arthritis (80), whereas elevated IGF-1 plasma levels are correlated with a declined risk for hypertension incidence in non-diabetic women patients (81).

IGF-1 has been linked to changes in cardiac structure. IGF-1, in particular, stimulates collagen type I synthesis in VSMCs and its extracellular accumulation (82). Moreover, IGF-1 could moderate the myocardium structure since it inhibits apoptosis in hypoxic cardiomyocytes (47). Despite this, there is no conclusive evidence that IGF-1 can modify cardiomyocytes in healthy heart tissue in addition to its observed overexpression (75, 83).

### 2.2 MiRNA-mediated regulation of IGF-1 and IGF-1R expression

Several studies have reported miRNAs-mediated epigenetic regulation of the expression and secretion of IGF-1 and its receptor, IGF-1R. MiRNAs are small, single-stranded RNAs that post-transcriptionally alter the expression of target genes (84, 85) and typically exert their regulatory effects in a context-dependent manner by binding to cis-elements located in the 3’ untranslated region (3’UTR) of target protein-coding mRNAs. In some cases, miRNAs may bind to 5’UTR or coding regions, affecting mRNA stability and degradation (85, 86). The expression of as many as 1/3 of the genes in the human genome is regulated by miRNAs. Several thousands of miRNAs have been identified so far, representing one of the most abundant gene expression regulators with a role in almost every biological process in multicellular organisms (87, 88). A single miRNA hypothetically may bind to more than a hundred target mRNAs, and several miRNAs can cooperate to finely tune the expression of the same transcript (89, 90).

So far, several miRNAs have been implicated in regulating IGF-1 expression and signaling (91). miR-320 was demonstrated to affect IGF-1 expression and insulin sensitivity in adipocytes by modulating the insulin signaling pathways (92). miR-126, whose levels are significantly decreased in multiple tissues in type 2 diabetes mellitus (T2DM), has also been reported to affect the expression of IGF-1 and IGF-2 (93). MiR-133a stimulated IGF-1R expression by prolonging the IGF-1R mRNA half-life. In atherosclerosis induced by apolipoprotein-E deficiency, decreased miR-133a expression is associated with lower IGF-1R levels and suppressive VSMC growth. Administration of miR-133a precursor increased IGF-1R levels and promoted IGF-1-induced VSMC survival and growth (94). In addition, a specific miRNA may exert multiple regulatory effects by binding to several targets of metabolic signaling pathways, illustrated by the example of let-7 that binds to 3’UTR regions of INSR, IRS-2, and IGF-1R and regulates their expression (95). Also, in cardiac and skeletal muscles, miR-1 controls the expression of both IGF-1 and IGF-1R (96). miR-143-3p is significantly upregulated in the serum of T2DM patients and various tissues of obese mice, such as skeletal muscle, pancreas, and heart. miR-143-3p was reported to regulate the IGF-2R receptor and may contribute to insulin resistance observed during MetS (97).

## 3 Effects of IGF-1 in physiological conditions

The classical view of IGF-1 as an essential growth factor is expanded by findings of its significant metabolic effects whereby IGF-1 stimulation of its receptor serves as a signal that notifies cells about the availability of adequate nutrients, thus coordinating protein, fat, and carbohydrate metabolism in various cell types. IGF-1 stimulates protein synthesis *via* the PI-3 kinase pathway in tissue cell culture. IGF-1 receptor activation is followed by a cascade of reactions starting with tyrosine kinase-mediated phosphorylation of insulin receptor substrate-1 (IRS-1) (98). IRS-1 is an adaptor protein that provides a binding site for PI-3 kinase, whose activation stimulates Akt (**Figure 2**). Consequent TSC-2 suppression and mTORC1 complex activation stimulates phosphorylation of p70S6 kinase and 4E-BP1 translational repressor. AMP kinase activated by nutrient restriction modulates this process by phosphorylating serine residue 794 on IRS-1, which prevents its activation and leads to the inhibition of PI-3 kinase and IGF-1-stimulated protein synthesis (99). IGF-1 also stimulates amino acid transport and protein synthesis in skeletal muscle while inhibiting protein breakdown (100, 101). IGF-1 induces skeletal muscle hypertrophy by blocking the transcriptional upregulation of the ubiquitin-ligases MuRF1 and MAFbx (also called atrogin-1), which serve as key mediators of skeletal muscle atrophy (101). This finding is supported by research demonstrating that genetically altered mice expressing atrogin complex constitutively show resistance to the anticatabolic effects of IGF-1 (101). Another study reported that IGF-1 partially antagonizes the cytokines activated in catabolic states that initiate muscle breakdown through MURF1 and MAF box induction (102). IGF-1-mediated inhibition of MAFbx upregulation by treatment reduces proteasome formation and targeting of proteins for degradation, thus lowering the rate of catabolism (103).

It has been proposed that IGF-1 may be a primary factor that maintains protein synthesis during intervals between meals, whereas insulin is a major factor that stimulates skeletal muscle anabolism following meal ingestion (104). This is supported by findings that IGF-1 treatment stimulates protein synthesis while having little effect on proteolysis and catabolism in healthy volunteers (105). However, IGF-1 can suppress proteolysis at high concentrations, even in normally fed subjects. In contrast, insulin inhibits proteolysis in muscle tissue at very low concentrations, and high concentrations are required to stimulate protein synthesis.

IGF-1’s ability to modulate insulin and GH actions allows it to affect carbohydrate metabolism. Specifically, IGF-1 reduces serum GH levels and affects insulin suppression of gluconeogenesis in the liver. Furthermore, increasing free fatty acid uptake in muscle IGF-1 indirectly enhances hepatic insulin action (106). GH was found to stimulate the PI-3 kinase p85 subunit synthesis (107), which leads to the suppression of p110 subunit activity and results in decreased insulin action (108). IGF-1 has been shown to stimulate glucose transport into muscle *via* IGF-1 receptors or insulin/IGF-1 hybrid receptors, and a high concentration of free IGF-1 has been shown to suppress gluconeogenesis in mice directly (109–111). Deletion of the insulin receptor in a mouse model showed decreased blood glucose levels in response to IGF-1 (112), whereas *in vivo* reduction of serum IGF-1 by 80% impaired glucose tolerance (113). Thus, IGF-1 enhances insulin sensitivity and decreases blood glucose, but its primary effects are increasing fatty acid oxidation in muscle, leading to the decreased hepatic free fatty acid influx and increased insulin suppression of liver glucose output (104).

IGF-1’s role in stimulating the uptake and oxidation of free fatty acid in skeletal muscle was confirmed by *in vivo* experiments with transgenic mice overexpressing a dominant-negative IGF-1R in skeletal muscle (MKR mice). These mice showed a significant decrease in glucose uptake when stimulated with either insulin or IGF-1, indicating a loss of function of both the IR and IGF-1R. Furthermore, MKR mice were insulin-resistant, had high serum and tissue lipid levels, and developed diabetes at a young age (114). In another study, MKR mice were crossed with mice overexpressing a fatty acid translocase, CD36, in skeletal muscle (115). Normalization of the rate of fatty acid oxidation in skeletal muscle and normalization of hyperinsulinemia, hyperglycemia, and significant improvement in hepatic insulin sensitivity was observed in the double-transgenic MKR/CD36 mice (115). These findings suggest the central role of IGF-1 in skeletal muscle fatty acid transport. They are also supported by the finding that long-term administration of recombinant IGF-1 in humans with GH receptor deficiency is associated with decreased fat mass, increased lipolysis, and a faster rate of lipid oxidation (116).

## 4 Effects of IGF-1 in pathophysiological conditions

It is extensively documented that IGF-1 exerts profound effects on CV and metabolic disorders. IGF-1 modulates CV function by stimulating angiogenesis (117, 118) and promoting anti-apoptotic and anti-inflammatory actions (119, 120). Also, IGF-1 activates nitric oxide synthase (NOS) *via* Akt-catalyzed phosphorylation, which stimulates NO production in ECs and VSMCs, leading to improved cardiac contractility (121). Furthermore, several studies suggest that IGF-1 may indirectly affect the CV system by increasing insulin sensitivity (113, 122–124). In the context of metabolic disorders, it has been shown that IGF-1 ameliorates insulin sensitivity (123, 124).

Moreover, perturbed IGF-1 levels are associated with insulin resistance, glucose intolerance, increased T2DM risk and cardiovascular morbidity and mortality (125, 126). IGF-1 independently correlates with coronary microvascular impairment, suggesting the crucial role of this growth factor in the increased CV risk associated with acromegaly (127). Also, a recent study reports profoundly reduced serum/plasma IGF-1 levels in patients with obstructive sleep apnea-hypopnea syndrome (OSAHS), a disease with severe CV and metabolic consequences that result in increased mortality of comorbid conditions such as coronary heart disease and cardiac failure (128). The following sections summarize the major findings concerning IGF-1 levels and pathophysiological conditions such as atherosclerosis, CAD, PAD, stroke, hypertension, and diabetes.

### 4.1 IGF-1 and CVD

The imbalance between pro- and anti-atherogenic factors results in atherosclerosis development. Numerous cell types, including inflammatory cells (macrophages), VSMs, and ECs, are directly or indirectly involved in the pathogenesis of atherosclerosis. Their byproducts promote endothelial dysfunction and accelerate the atherosclerotic process (129–131). IGF-1, one of the byproducts, exhibits pro- and anti-atherogenic properties (14). The risk of atherosclerotic CV disease was lower in healthy persons with high free IGF-1 levels in the blood (22, 132). Accordingly, the risk of developing acute coronary syndrome has also been linked to low IGF-1 levels (53, 133, 134). Lower IGF-1 concentrations in individuals with acute myocardial infarction were linked to a higher incidence of post-myocardial infarction consequences and a worse prognosis (133, 135). A study by Stavropoulou et al. showed that the serum IGF-1 levels in infarcted rats were initially decreased (24 h up to 1 week) and remained unaltered during the late postinfarction period (4 to 8 weeks). Interestingly, this study reported significant upregulation of IGF-1 expression in rat myocardium after coronary artery infarction. The fact that an increase in serum IGF-1 levels did not accompany it suggested that increased IGF-1 expression serves the needs of rat myocardium locally, participating in the postinfarction repair processes (136).

The preferred laboratory models for atherosclerotic diseases are murine and pig models. Murine atherosclerotic models are primarily used at the initial phase of pre-clinical research, as opposed to pig models, which are reserved for later experimental stages (i.e. testing the pharmacokinetics, pharmacodynamics and side-effects of experimental anti-atherosclerotic drugs). Pigs were found to have lipid profiles and atherosclerotic coronary artery involvement that were very similar to humans (137).

The pre-clinical studies are mostly based on animal mechanical injury models demonstrating a link between increased IGF-1 and neointimal proliferation (138–141). IGF-1 inhibition inhibits neointimal proliferation and could be clinically used to treat restenosis after coronary artery angioplasty (142).

There has been little research into the role of IGF-1 in hypercholesterolemic animal models of atherosclerosis with no mechanical injury (143). In general, such animal models revealed that low circulating IGF-1 levels are associated with more extensive atherosclerosis (144), whereas high circulating IGF-1 levels are not (9). Sukhanov et al. demonstrated that IGF-1 infusion reduced atherosclerotic lesion extension (aortic root plaque area by 30%), vascular oxidative stress, inflammation, and atherosclerotic plaque macrophage infiltration in a murine Apoe-/- model (145). Shai et al. demonstrated that chronic IGF-1 overexpression in smooth muscle cells (SMCs) did not increase overall plaque extensiveness and exhibited features of stable plaques in Apoe^-/-^ mice fed a Western diet (146). Interestingly, IGF-1’s atheroprotective effects are largely independent of SMCs (146). IGF-1R deficiency in Apoe^-/-^ mice macrophages increases atherosclerotic extensiveness and destabilizes plaque stability by lowering SMCs and collagen content, according to the pleiotropic effects of IGF-1 on various vascular wall cells (119). IGF-1 signaling disruption determines macrophage activation into the pro-inflammatory M1 phenotype and reduces lipid efflux, promoting lipid accumulation in macrophages (119, 147). In contrast, Snarski et al. demonstrated in a macrophage-specific IGF-1 Apoe^-/-^ mouse model that macrophage-derived IGF-1 inhibited the expression of chemokine (C-X-C motif) ligand 12 (CXCL12), reduced monocyte recruitment to plaques, and favoured macrophage cholesterol efflux (60). Recombinant human IGF-1, given over 6 months at a dose FDA-approved for long-term treatment of growth failure in children with severe primary IGF-1 deficiency, reduced coronary artery atherosclerosis and promoted a stable plaque phenotype in a pig model of familial hypercholesterolemia (FH) (148). IGF-1 suppression of systemic inflammation and oxidative stress provides such effects. In plaques, IGF-1 suppressed gene expression of FOS/FOSB transcription factors, CXCL14, and matrix metalloproteinase 9 (MMP9), according to a spatial transcriptomics study (148).

The clinical indicator of atherosclerosis across the body and in the brain is the common carotid artery intima-media thickness (CC-IMT). Extensive research has shown a strong association between CC-IMT and IGF-1 levels (52, 149–152). Intriguingly, Sirbu et al. (153) proposed that elevated IGF-1 levels might promote plaque stability in advanced atherosclerosis while stimulating smooth muscle hyperplasia in early atherosclerosis. The same study reported that the measure of insulin resistance, HOMA-IR, and total IGF-1 z-score, is associated with increased CC-IMT (153). Also, a positive correlation between CC-IMT and serum IGF-1/IGFBP-3 ratio was reported (52, 150). Furthermore, a negative association of IGF-1 with CC-IMT was observed only in patients with low vitamin D levels (152) and was supported by findings of a comparative study of obese individuals and healthy controls showing that IGF-1 protects against CC-IMT when serum vitamin D levels are low (154). Recent findings show that vitamin D3 treatment activates the IGF-1 promoter and enhances IGF-1R signaling, accompanied by enhanced mesenchymal stem cells-induced angiogenesis and increased vascularization *in vitro* and *in vivo* (155).

The PRIME prospective cohort study investigating the relationship between IGF-1 and CAD in 10,600 patients reported that IGF-1 levels negatively correlated with age, markers of inflammation, waist circumference, and tobacco consumption (156). The study revealed that participants with the acute coronary syndrome had significantly lower baseline IGF-1 levels, and those in the highest quartile for IGF-1 levels had a 55% lower relative risk of myocardial infarction (156). Several other studies confirmed a relationship between elevated IGF-1 levels and a decreased prevalence or incidence of CAD (156–158). Additionally, it was found that individuals with early-onset CAD had considerably lower IGF-1 levels (157).

Increased IGF-1 levels protect against ischemic strokes, as evidenced by the lower prevalence or incidence of ischemic stroke (54, 159, 160). For instance, the lowest ischemic stroke incidence was observed for the patients in the highest quartile of circulating IGF-1 (232 ± 41.04 ng/ml) according to a prospective study based on observational data from 757 participants of the Framingham Study (54). This correlation was especially pronounced in patients with insulin resistance and subjects with the highest waist/hip ratio, leading to the conclusion that low circulating IGF-1 levels may be associated with an increased risk of ischemic stroke in obese and diabetic patients due to higher insulin resistance (54).

Vasodilation and reduced vasoconstriction, anti-apoptotic and pro-survival effects on cardiomyocytes and vascular bed cells, tissue remodeling effects, and stimulation of cardiac development and contractility represent positive effects of IGF-1 on the CV system (**Figure 2**) (14). Even though the majority of research demonstrates a clear correlation between low serum IGF-1 levels and an increased risk of CV diseases (15, 123, 161), specific conflicting findings can be ascribed to methodological problems in assessing total and free IGF-1 levels (161, 162). For instance, it was reported that the risk of CV events such as acute coronary syndrome was reduced in the presence of higher IGF-1 levels (156, 163). Furthermore, the fact that therapy with IGF-1 or its analogues had a favorable effect on patients with chronic CV diseases recognized the role of IGF-1 in the CV system (164, 165). In line with that, Rotterdam and Framingham’s studies revealed that IGF-1 levels are negatively linked with the incidence of CV events (166, 167). Based on a dose-response analysis, every 45 μg/mL increase in IGF-1 correlates with a 9% reduction in overall CV events risk for both genders (163). In addition, a longitudinal study in a large number of subjects prone to hypertension and CVD demonstrated a protective association between IGF-1 and hypertension, CV and all-cause mortality (168).

#### 4.1.2 IGF-1 and PAD

At present, there is limited availability of data regarding the potential association of IGF-1 with PAD (169). In a study by Urbonaviciene et al., serum IGF-1 and IGFBP-2 levels were measured in 440 patients with lower-extremity PAD, revealing an increased risk of CVD mortality was associated with an increase of 100 μg/l of baseline IGFBP-2 value. Furthermore, the study concluded that elevated IGFBP-2 levels in patients with PAD were independently associated with long-term CVD mortality. However, the addition of IGFBP-2 to a model containing conventional CVD risk factors did not improve the risk prediction of CVD mortality (169).

In another cross-sectional study, circulating IGF-1, IGFBP-3, and labile acid subunit (ALS) essential for maintaining normal serum IGF-1 and IGFBP-3 concentrations (170), were measured in PAD patients and healthy controls. In addition, IGF-1, C-reactive protein (CRP), IGFBP-3, and ALS were measured in blood from the aorta and femoral vein of the affected limb in a subset of patients subjected to peripheral angiography. PAD patients had decreased levels of IGFBP-3, and measurements in the affected limb revealed a negative correlation between CRP and IGF-1 venous-arterial difference, whereas a positive correlation between CRP and IGFBP-3 venous-arterial difference was observed. This study established a connection between systemic levels of IGF axis components and the presence of PAD, suggesting that inflammation of the affected limb in PAD patients affects the transfemoral IGF-1 and IGFBP-3 concentrations (171).

#### 4.1.3 IGF-1 and hypertension

IGF-1 mediates the regulation of blood pressure by stimulating endothelial nitric oxide synthase (eNOS) activity and consequent nitric oxide (NO) production in ECs that promotes vasodilatation and increases blood flow (172–174). In rats and mice with genetic IGF-1 deficiency, an elevated mean blood pressure is observed, affirming the IGF-1’s role in blood flow and pressure regulation (76, 174, 175). Increased circulating IGF-1 levels in hypertensive subjects were shown to promote adaptive structural alterations such as ventricular hypertrophy and vascular remodeling (176). In addition, a recent study reports an association between plasma IGF-1 levels and intraventricular septal thickening (177). The reduced vasodilation response to IGF-1 1 was reported in hypertension (178, 179) which may exert a considerable effect in the development of arterial hypertension. It has been suggested that increased matrix metalloproteinase (MMPs) activity in hypertension results in the proteolytic cleavage of the extracellular IGF-1R α subunit and the observed lack of receptor sensitivity (19). Several studies support this notion by showing that increased proteinase activity plays a vital role in organ damage and the loss of cellular function in hypertension (180–183).

However, literature analysis of human studies shows conflicting reports on the association between blood pressure regulation and IGF-1 levels. For instance, several studies on a smaller number of patients showed higher IGF-1 levels in hypertensive patients compared to normotensive subjects (184–187), whereas several cross-sectional studies indicated a neutral relationship between blood pressure and IGF-1 (52, 134, 188–190). Nevertheless, several more recent large cross-sectional studies reported a significant inverse correlation between blood pressure and IGF-1 (123, 191–196). Also, prospective studies confirm the inverse association of IGF-1 with systolic blood pressure and significantly reduced risk for incident hypertension in non-diabetic female subjects (81).

#### 4.1.4 IGF-1, diabetes, and hyperinsulinemia

A link between decreased circulating IGF-1 and MetS components, such as dyslipidemia and DM, has been observed (15, 161) but remains questionable due to conflicting findings in the literature. It has been established that IGF-1 improves insulin sensitivity (123, 124), and higher IGF-1 levels are associated with fasting insulin levels and insulin resistance (197). The risk of developing T2DM and CAD was discovered to be genetically predisposed in people with elevated serum IGF-1 levels (197). More specifically, persons with insulin levels above the median and high levels of free IGF-1 have a higher chance of developing T2DM, whereas those below the median have a lower risk (198).

On the other hand, one study found no link between IGF-1 levels and T2DM (199). Additional longitudinal studies provided evidence of a strong association between increased incidence of insulin resistance and T2DM in subjects with either low or high IGF-1 serum levels (200, 201). The findings suggested that the association of IR and T2DM with low IGF-1 levels is due to the insulin-like actions of IGF-1 that promote hypoglycemia and IGF-1 suppression of growth hormone secretion, which causes insulin resistance (202, 203).

## 5 Therapeutic interventions that target the IGF axis

Due to its central role in IGF signaling, the IGF-1R has been studied as a target for therapeutic interventions using several strategies that were developed for clinical studies, such as anti-IGF-1R antibodies, IGF-1/2 neutralizing antibodies, nucleic acid-based approaches, small molecule tyrosine kinase inhibitors (TKIs), and IGF ligand TRAPs. Notably, most studies were performed in the context of potential novel treatments for various cancers, and the effects of such therapeutic approaches on cardiovascular and metabolic diseases are still not sufficiently reported in the literature. IGF-1R was the first tyrosine kinase targeted by a monoclonal neutralizing antibody (alpha-IR-3) that blocked the receptor binding domain (204). However, concerns about toxicity resulting from potential INSR co-inhibition halted further clinical evaluation of this approach (205). Nucleic acid-based approaches such as antisense oligonucleotides (ASOs), small interfering RNAs (siRNAs), and dominant-negative receptors successfully blocked IGF-1 signaling *in vitro* and *in vivo*. ASO-mediated downregulation of IGF-1 or IGF-1R showed promising inhibitory effects on the IGF axis *in vivo* (206, 207). For instance, chronic intravenous administration of IGF-1R ASO in spontaneously hypertensive rats (SHR) was employed to study the effects of a functional deficit in IGF-1 signaling (208). IGF-1 ASO administration decreased IGF-IR expression in conductance and resistance blood vessels, reduced aortic IGF-1R density, and angiotensin II type 1 receptor expression (208).

RNA interference (RNAi) technology based on the use of short (20–25 bp) double-stranded siRNAs that recruit the RNA-induced silencing complex (RISC) can be programmed to target virtually any nucleic acid sequence. Typically, siRNAs post-transcriptionally silence target genes *via* mRNA degradation, but they can also interact with the transcriptional machinery to induce transcriptional repression and may participate in epigenetic modifications (209, 210). Bohula et al. designed an IGF-1R siRNAs that efficiently silenced the *IGF1R* gene (211). However, the potential clinical use of such siRNA molecules requires their stabilization and improved delivery to improve their effectiveness upon *in vivo* administration (212).

Another molecular approach is based on the expression of the dominant-negative IGF-1R receptor (dnIGF-1R), which makes a complex with a wild-type half receptor and can bind a ligand but lacks kinase activity, thus blocking the function of endogenous IGF-1R. Since IGF-1R is a heterotetramer in which one ligand molecule binds into a pocket formed by two IGF-1R α subunits (213, 214), dnIGF-1R were constructed by expression of IGF-1R residues 1–950 that are present at the cell surface or residues 1–486 that encode soluble receptor (215–217). Similarly, dominant negative IGF-1R inhibition was achieved using an integrin binding-defective mutant of IGF-1 (218). Further advancement in IGF-1 signaling inhibition was achieved by the development of small molecule tyrosine kinase inhibitors (TKIs) that block IGF-1R kinase activity (219). However, these TKIs also inhibited INSR due to a high level of sequence homology between IGF-1R and INSR-A/B kinase domains (220–222) which may lead to adverse effects such as hyperinsulinemia (223). Also, the short half-life of TKIs may result in transient inhibition of target receptors, and maintenance of an efficient dosing regimen is associated with a risk of toxicity (224–226).

In the specific context of IGF-1-based therapeutic approaches for the treatment of metabolic diseases, it should be mentioned that recombinant human IGF-1 (rhIGF-1) has been studied as a potential treatment for diabetes. There is evidence that rhIGF-1 administration improved glycemic control but was associated with severe adverse effects such as diabetic retinopathy aggravation (227). Although this approach to diabetes treatment has been abandoned, IGFBP-1 and -2 have recently emerged as potential targets for insulin sensitivity modulation and treating diabetes and obesity (202). Another therapeutic approach with promising potential has been recently proposed by Wang and colleagues, who reported that significant elevation of IGF-1 levels could be achieved by combination treatment with tadalafil (TAD) and hydroxychloroquine HCQ. TAD is a phosphodiesterase 5 inhibitor that exerts cardioprotective effects against ischemia/reperfusion (I/R) injury in diabetic mice, whereas HCQ is an antimalarial and anti-inflammatory drug that reduces hyperglycemia in diabetic patients. The observed increase in insulin and IGF-1 levels upon TAD+HCQ treatment resulted in the activation of the Akt/mTOR signaling pathway. As a result, it was proposed that concurrent TAD and HCQ treatment could be a readily available novel pharmacotherapeutic approach for protection against myocardial I/R injury in T2DM (228).

Sodium-glucose transporter-2 inhibitors (SGLT-2is) and glucagon-like peptide-1 receptor agonists (GLP-1RAs) exhibit multiple metabolic and CV effects that have a positive impact on diabetic state and metabolism in general, such as reduction of hyperglycemia, fat mass and bone remodeling, natriuresis weight loss, and anti-atherosclerosis (229). Increasing evidence indicates the involvement of the IGF axis in pleiotropic responses elicited by SGLT-2is and GLP-1RAs. For instance, GLP-1RAs stimulate pro-survival responses in tissues where the IGF-1/IGF-1R plays an important role, such as pancreatic β-cells and the heart. SGLT-2is effect on the IGF axis is poorly documented, but it has been speculated that they promote increased ketogenesis mediated by elevated circulating GH levels (229). The association of SGLT-2is and GLP-1RAs with the IGF axis represents an exciting novel pharmacological strategy for the modulation of IGF-1 levels, which requires further validation for potential application in the treatment of CVD and metabolic diseases (229).

## 6 Conclusions

IGF-1 has numerous beneficial and protective effects on the CV system, including anti-apoptotic and pro-survival effects on cardiomyocytes and vascular bed cells, tissue remodeling effects, cardiac development and contractility stimulation, vasodilation and decreased vasoconstriction. Although the majority of available research data demonstrate a correlation between low serum levels of IGF-1 and an increased risk of CV diseases, conflicting findings can be ascribed to methodological problems in assessing total and free IGF-1 levels and the lack of standardization of the IGF-1 assays used in different studies. Therefore, future efforts should focus on introducing measures to standardize methods for measurements of total and free serum IGF-1 to facilitate the interpretation of correlations between IGF-1 and different parameters of cardiovascular and metabolic diseases. The intriguing possibility of targeting the IGF axis for treating cardiovascular and metabolic disorders remains uncertain due to adverse effects observed in pre-clinical and clinical studies. Recent findings regarding miRNAs-mediated regulation of IGF-1 expression open new avenues for designing and developing nucleic acid-based therapeutic agents that can finely tune IGF-1 levels and confer protective effects in cardiovascular and metabolic disease-associated conditions.

## Data availability statement

The original contributions presented in the study are included in the article/supplementary material. Further inquiries can be directed to the corresponding author.

## Author contributions

MM- wrote the article; ZG -wrote the article; JR-wrote the article, ME - wrote the article, XG - wrote and critically reviewed the article and EI - wrote and critically reviewed the article. All authors contributed to the article and approved the submitted version.
